# Trusted Data Spaces as a Viable and Sustainable Solution for Networks of Population-Based Patient Registries

**DOI:** 10.2196/34123

**Published:** 2023-01-13

**Authors:** Nicholas Nicholson, Sandra Caldeira, Artur Furtado, Ciaran Nicholl

**Affiliations:** 1 European Commission Joint Research Centre Ispra Italy; 2 European Commission Directorate General for Health and Food Safety Luxembourg Luxembourg

**Keywords:** population-based patient registries, trusted research environments, registry network model, data federation, cancer registries, noncommunicable diseases

## Abstract

Harmonization and integration of health data remain as the focus of many ongoing efforts toward the goal of optimizing health and health care policies. Population-based patient registries constitute a critical element of these endeavors. Although their main function is monitoring and surveillance of a particular disease within a given population, they are also an important data source for epidemiology. Comparing indicators across national boundaries brings an extra dimension to the use of registry data, especially in regions where supranational initiatives are or could be coordinated to leverage good practices; this is particularly relevant for the European Union. However, strict data protection laws can unintentionally hamper the efforts of data harmonization to ensure the removal of statistical bias in the individual data sets, thereby compromising the integrated value of registries’ data. Consequently, there is the motivation for creating a new paradigm to ensure that registries can operate in an environment that is not unnecessarily restrictive and to allow accurate comparison of data to better ascertain the measures and practices that are most conducive to the public health of societies. The pan-European organizational model of cancer registries, owing to its long and successful establishment, was considered as a sound basis from which to proceed toward such a paradigm. However, it has certain drawbacks, particularly regarding governance, scalability, and resourcing, which are essential elements to consider for a generic patient registry model. These issues are addressed in a proposal of an adapted model that promises a valuable pan-European data resource for epidemiological research, while providing a closely regulated environment for the processing of pseudonymized patient summary data on a broader scale than has hitherto been possible.

## Context

The World Health Organization (WHO) defines public health as “the art and science of preventing disease, prolonging life and promoting health through the organized efforts of society.” Surveillance of population health and well-being is listed as the first point in WHO’s list of 10 essential public health operations [1].

In practice, the task of monitoring health status is fraught with difficulty. Health data need to be collected from many different points, raising challenges in data harmonization, data contextualization, and data quality. Depending on the type of public health infrastructure, there may additionally be different regional practices that compound these types of problems. Moreover, the introduction of strict data protection laws adds further layers of complexity in linking data, which is an essential part of the process. A wide range of endeavors has been undertaken to align health data, and a certain level of progress has been made through international collaboration on many different aspects of the underlying challenges. However, within Europe, fragmentation of data still persists owing in part to a rich tapestry of different types of national health care infrastructure in place.

In the field of population health monitoring, population-based patient registries are valuable assets in the surveillance and control of disease. Registries expend substantial effort to ensure accuracy and completeness of data in a given population to limit statistical bias [2]. Moreover, registry data are structured and generally well described using standardized coding classifications, which have positioned registries as key epidemiological resources [3,4]. Although registries are relatively autonomous in the population they cover, an extra dimension in their usefulness and value is created by the integration and comparison of data across registries. Combined registry data use allows studies with large cohorts of patients, thus leading to deep analyses and enhanced knowledge [5,6]. Despite the potential considerable savings in resources and benefits to patients that integrating registry data can bring [7,8], there have been few concerted efforts to support or facilitate such initiatives in a sustainable manner. Moreover, increasingly strict data protection regimes can lead to significant administrative overheads in the collection and processing of pseudonymized data, further exacerbating the challenges.

Epidemiological research is particularly affected by strict data protection paradigms that do not generally differentiate the needs of accessing personal data for data linkage purposes from that for studies on specific individuals. Epidemiology requires data linkage solely to derive the patient cohorts of interest (rather than to conduct studies on specific individuals). Moreover, to ensure unbiased intercomparisons of national data, the necessary data validation techniques require access at least to pseudonymized data, which are also considered as sensitive data by the strict data protection paradigms. In view of the current difficulties and barriers confronting initiatives to monitor disease at a supranational level for the purposes of informing and coordinating strategies for control and prevention of disease at the European Union (EU) level, new mechanisms are required to facilitate the necessary access to data without conflicting with the core intent of EU’s general data protection regulation (GDPR) legislation.

EU Member States have committed to the United Nations’ sustainable development goals to reduce premature mortality owing to noncommunicable diseases (NCDs) [9], and the European Commission (EC) is addressing the rise in numbers of European citizens experiencing the more prevalent chronic diseases [10]. In particular, EC continues to provide support to the work of cancer registration through the European Network of Cancer Registries (ENCR) to derive statistical indicators used to steer EU policies and coordinate Member States’ activities for tackling the cancer burden. EC has also funded several projects among the Member States to further the availability of indicators for other diseases [11-13] and supported registry networks via specific project-related activities [14]. However, these initiatives are of relatively short duration, and EU funding cannot replace national support for recurrent activities within country remits. What is missing is a long-term perspective about patient registry networks leading to a sustainable solution. After the conclusion of the joint action initiative on cross-border patient registries in 2014 [14], the European Medicines Agency launched several follow-on initiatives including the creation of an inventory of patient registries and a draft guideline on registry-based studies, which were open to public consultation [15]. Although the aim is to create an EU-wide framework for patient registries, the framework is with reference to facilitating general, open collaboration between the various stakeholders rather than specifically addressing models for integrating registries’ data. Although patient registries in Europe are generally willing to establish supranational networks [16,17], for the most part, they struggle to find the resources and instruments to do so, and an EU-coordinated sustainable and scalable model of patient registry networks would surmount many difficulties. In addition, such a model could scale to other regions of the world in which there are agreements between countries for tackling common public health issues, thus further increasing the potential of the approach.

## A Missing Infrastructure

Despite the acknowledgment that policy and decision-making processes should be based on evidence and supported by adequate health information systems, Bogaert and Van Oyen [18] observed that “there is no single comprehensive EU-wide public health monitoring system or health information system that allows policy-oriented research or advice.” Although indicators of NCDs have been, and continue to be, estimated from various sources including hospital discharge records, death certificates, health insurance claims data, and health surveys, the quality of such indicators can be extremely variable. This is particularly the case for health insurance data that can lead to biased coverage of the population where there is no universal national health insurance system. For these reasons, insurance data tend to be used for evaluating and assisting health care planning rather than for generating health statistics [19]. Other difficulties arise from the different disease coding systems used and even different interpretations of codes within the same coding system. Moreover, health surveys have limited sample sizes and tend to miss illnesses with high fatality rates [19]. Although in the absence of any systematic collection of data, these estimated processes play an important role, they also place onerous demands on the institutions involved when conducted on a periodic basis, and there will be a trade-off between the resources required and the cost-benefit of the indicators depending on their quality and veracity. Many challenges facing such initiatives are summarized in the Eurostat report on pilot studies performed for collecting morbidity statistics in EU [19].

Regarding the establishment of a dedicated EU health information system, Rosenkötter et al [20] identified the key features expected from an EU health information system based on a series of interactions with stakeholders from international institutions, national ministries, public health authorities, universities, and EU cofunded projects. In a ranking of 10 features, the most important one was considered to be a permanent and sustainable system (in terms of content, infrastructure, and resources), followed by quality of data and information. The authors note that in spite of the acknowledged relevance of public health surveillance and the numerous requests for a comprehensive EU health information system, there seem to have been considerable barriers to hinder its realization. They cite various possible factors such as the data collection processes through different and fragmented national health information systems and the concerns of oversimplified rankings in data comparisons; the specific types of issues related to both these factors have been described by Tijhuis et al [21]. An even more fundamental problem identified by WHO is that “measuring health is conceptually and technically complex, requiring statistical, public health and biomedical knowledge and expertise unique to each disease or programme area” [22], and therefore dependent upon coordination and integrated strategies across many different entities.

Although population-based patient registries may have variable coverage and comparability [19], they nevertheless provide a well-established and systematic data collection process, with data often stretching back many years. Additional value is obtained through their collaboration within supranational networks, as these provide the means to harmonize indicators via agreement on data alignment, validation, and cleaning processes. This is essentially the approach followed by ENCR in the collection and derivation of European cancer indicators.

Stakeholder discussions have since gained momentum toward the establishment of the European Health Data Space (EHDS) [23], which foresees an infrastructure that facilitates access to health data across EU. It is not yet clear whether the envisaged solutions underlying the implementation of EHDS will be sufficient to provide the level of service needed for an accurate comparison of health indicators across Member States. Many challenges still need to be addressed, including the timely provision of data, ensuring the prerequisite quality and harmonization of data, and issues related to data linkage. The extent of these difficulties can be appreciated from the processes followed by ENCR to ensure the harmonization and quality of cancer indicators for meaningful comparison across national boundaries. In view of the underlying verification and validation processes to ensure harmonized quality indicators, it is likely that ENCR will need to furnish EHDS with precomputed cancer indicators rather than the converse scenario, whereby European cancer indicators can be computed from data made available by EHDS. In this regard, EHDS will not necessarily circumvent the need for a network of cancer registries (CRs) or any other supranational registry network providing harmonized indicators at the EU level. Therefore, agreeing with a sustainable approach toward the operation and resourcing of such networks remains as an important objective.

The advantage of a registry network is precisely that it consists of a network of experts that allows it to function as more than a mere data collection and validation point. The WHO’s Health Metrics Network categorizes a health information system into 6 components (resources, indicators, data sources, data management, information products, and dissemination or use) [22], and these components stand to be addressed more holistically in entities such as registry networks, which also allow for supranational coordination and harmonization. Moreover, given that registries collect hospital discharge records and outpatient records, many of them either do or could collect comorbidity data that are considered to be necessary for a comprehensive picture of public health [19].

## Building on the Model of ENCR

### Overview

To gauge the practical feasibility of sustainable models enabling the collation of accurate and harmonized indicators for intercomparison at the supranational level, experiences and lessons can be drawn from existing, well-established networks. In this regard, ENCR serves as a prime example. We present the network in terms of how it is organized and how it currently operates, especially in view of the associated advantages and disadvantages.

## ENCR Organizational Structure

The members of ENCR are national or local CRs. They are not always public authorities. The participation of registries in ENCR is voluntary and has been promoted by EU initiatives since the 1990s. The funding for participation in the network is obtained from the budgets of each registry and from EU cofinancing.

The organizational structure of ENCR consists of a steering committee, with a 3-year rolling mandate. Members are either elected by the member registries or nominated by some of the participant bodies. The steering committee is responsible for prioritizing the issues facing the network and establishing working groups comprising individuals with specific skills or experience from the individual registries. Examples include review and revision of coding recommendations, agreement of data validation rules, and harmonization of variables extending the common data set. The steering committee is assisted by a secretariat that provides administrative and technical support.

The secretariat was initially provided by WHO’s International Agency for Research on Cancer, which played an instrumental role in the formation of the network in 1990 under EC’s Europe Against Cancer action plan. In 2012, the secretariat was transferred to EC’s Joint Research Centre. ENCR has been essential for coordinating the periodic collection of data for deriving the statistical indicators used at the EU level to compare cancer incidence, mortality, and survival and their trends over time for different types of cancer. The indicators are publicly available on the European Cancer Information System (ECIS) website [24], which is the reference point for monitoring and projecting the burden of cancer in Europe.

## Challenges Facing the ENCR Model

### Overview

Ensuring the accuracy of comparable cancer data from many different registries is a nontrivial process. The organizational landscape of CRs is extremely heterogeneous across Europe, and the complexity of the situation can be gauged by the sheer number of registries constituting ENCR (>150 individual CRs). Registries can be nationally based, regionally based, or even metropolitan, and Member State coverage is also quite variable [25]. Challenges relate to data curation, data interfaces, and lack of legal entity status for an informal network.

## Data Curation

**Figure 1:**
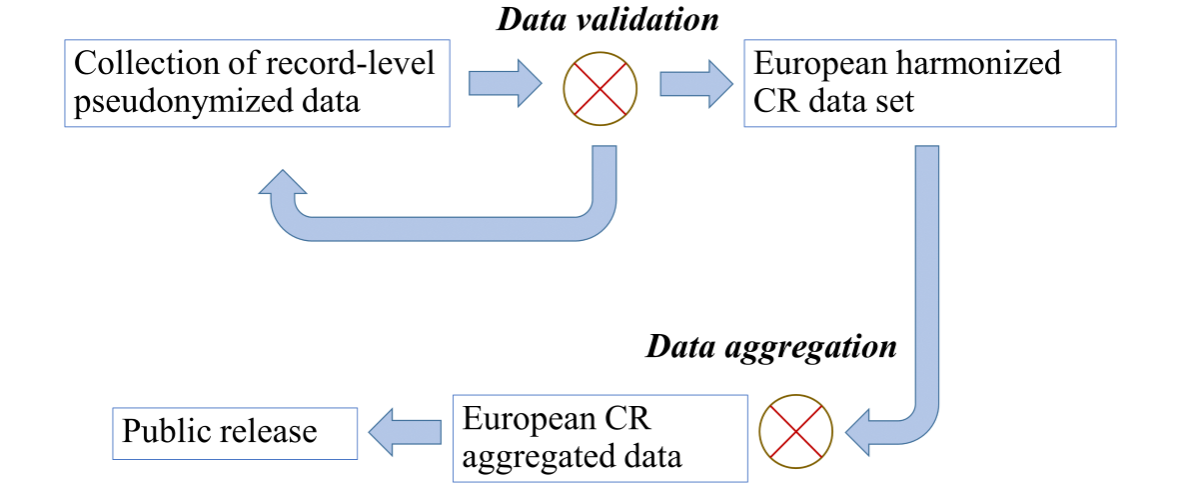
Summary of the centralized European cancer-registry data collection and cleaning process.

The collection of data from all CRs relies not only on the good will of the registries to provide their data within a given time frame but also on the legal contracts between the data collector and the data provider. First, the data are provided as pseudonymized patient summary records conforming to a template describing the ENCR common data set, which consists of harmonized variables fundamental for deriving the major statistical indicators. Then, the data are cleaned according to a set of predefined rules; the more basic checks relate to file formatting errors and ensuring that all the mandatory variables have been provided. The more intricate checks verify the accuracy of the variables’ data ranges, particularly regarding the values of other variables (intervariable checks). The data cleaning step may require several iterations with the registries until the data are considered to meet the required level of conformity with the data rules, which is necessary for national and international comparisons. Finally, the indicators are derived from the data on a per-registry basis and displayed at an aggregated level that removes the possibility of reidentification. Following a round of final checks with the registries, they are uploaded to the ECIS website. The process is summarized in Figure 1.

The processes for collecting, cleaning, and final verification add time delays beyond those already incurred by the CRs in collecting and verifying the data according to their own local processes and requirements. Once the aggregated data are made available at the European level, they are generally 4 to 5 years out of date. Forecasts, based on the historic data, are calculated to cover the intervening time lag; nevertheless, it stands to reason that any possible means of reducing the delays while not compromising the veracity of the data should be promoted. Delayed availability of data compromises their usefulness in feeding back in real time to health care policies, especially in monitoring the immediate effects of the introduction of cancer control initiatives, such as screening programs.

## Data Interfaces

Another major difficulty lies with the interfaces between the various independent entities involved in the data chain, especially in terms of data encoding and anonymization.

## Encoding of Data

**Figure 2:**
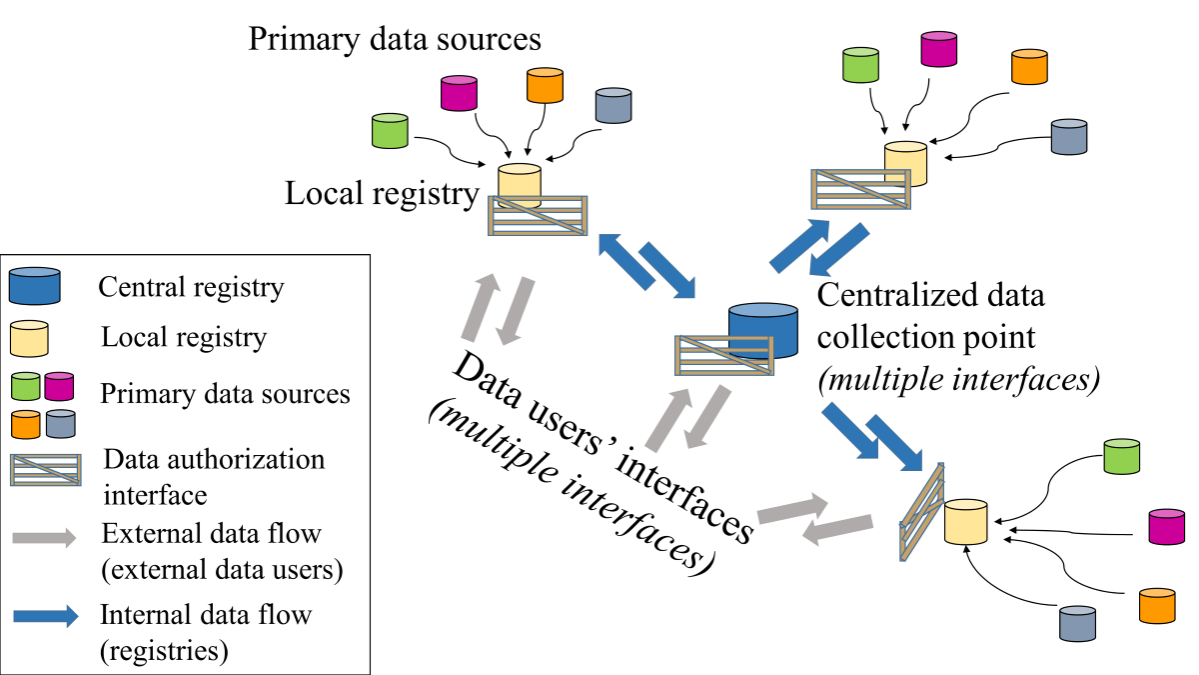
Multiple data interfaces exist in the common pan-European registry model. Each interface adds barriers to the type of data interaction allowed. In particular, the interface presented to general data users is complex on account of the lack of a single access point.

In primary health care settings, clinicians are busy and often may not have the time necessary to complete or verify the summary data that are forwarded to the registry. Medical secretaries entrusted with the work may themselves not have the necessary experience to verify the data or determine the correct codes to use from the case reports. If the data are not encoded accurately, the resulting inconsistencies tend to amplify the inefficiencies as they propagate through the downstream processes. Improved focus and attention given to ensuring the accuracy of the summary case reports at the initial point of encoding could save considerable expense and delay in correcting the data later. However, the same is true for all the interfaces, with each interface adding to the overall delay and inefficiency, as data have to be checked, verified, and corrected via dialogue between both sides of the interface. Figure 2 illustrates the multiple data interfaces in the current registry network scenario. Although these processes are in the domain of national health infrastructure, there are motives for establishing good practices in view of saving cumulative costs, both in terms of time and resources. Data reporting is often viewed as an unnecessary burden in the busy clinical environment, and further efforts could be undertaken to provide the results of data analyses back into the hands of the clinicians to help change such attitudes and demonstrate the practical advantages of accurate data reporting. In addition, measures could be implemented, such as training, data quality audits, or provision of standard templates encapsulating data quality and data semantic contexts following, for example, the model of Findable, Accessible, Interoperable, and Reusable data digital objects [26].

## Data Anonymization

Moreover, where the interface bridges different health care authorities, the transfer of pseudonymized data also falls under the data protection requirements of GDPR. Data requesters are required to state explicitly the intended purposes for the data, and these requests have to pass through various overseeing processes to ensure that the purposes are appropriate. The interfaces are further complicated between national boundaries where different legal interpretations and contexts may apply. The way in which GDPR has been enforced across different EU Member States since its introduction in May 2018 means that some registries are no longer able to provide even pseudonymized summary data. However, anonymized data cannot be used for verifying data consistency and bias. This development has profound consequences for the reliable intercomparability of the indicators currently provided in ECIS. Although initiatives are underway to federate the data cleaning processes, which would eventually remove the need to collect record-level data, it is not a straightforward task because there are many exceptional cases to handle and apply through a consistent approach. Moreover, merely federating the centralized entity’s processes does not tackle the broad and more important issues of leveraging the full value of the registries’ data (in a centralized data collection process, the focus tends to be on only the reduced subset of the core data variables).

## The Legal Entity Issue

The ease of acquiring data, at least from the perspective of recognized legitimacy of the data request, can be facilitated via a legal mandate. However, a legal mandate requires a data-requesting entity to be a legal entity, and although this does not pose any problems for individual registries, an informal network of registries is itself not a legal entity. The lack of legal entity status also confounds funding of the network or any financial transaction. This means that the network is fully dependent on the legal entity or entities that perform the administrative affairs of the network.

## Proposal for an Adapted Model

### Overview

The considerations mentioned in the previous section can be used to frame a proposal toward a more robust and efficient model by streamlining the processes, increasing coordination among the various entities, and providing the capacity to scale across other registry domains. Apart from cancer, other chronic disease domains receiving attention for more coordinated European initiatives include diabetes, cardiovascular disease, and chronic obstructive pulmonary disease. The need to increase the availability and access to NCD data for both policy and research uses has long been argued within Member State public health institutes and by experts and stakeholders in the field. However, important challenges remain before the provision of accurate, comparable data on key aspects can be realized—even of fundamental information such as the prevalence of diabetes, cardiovascular disease, or chronic obstructive pulmonary disease. Moreover, it would be particularly valuable to be able to track and derive geographical and temporal trends of epidemiological indicators (such as prevalence, incidence, mortality, and survival) for all the major NCDs and any comorbidities.

We argue that a paradigm shift with the potential for a significant increase in efficiency is achievable if all the superfluous external interfaces are removed. This could be realized by creating a trusted registry space, for example, similar to trusted research environments (TREs) being established in the United Kingdom for data-driven health research [27]. An environment of this nature would create a secure and trusted working space in which registries within a given disease domain could operate across regional and national boundaries.

## Pan-EU TREs

In TREs, researchers can only access the types of data agreed upon within their data sharing agreements. They are also prevented from downloading any record-level data and can only access the data within the TRE. Creating such an EU-wide environment on a registry domain basis would effectively unleash the full potential of registry data for the benefit of all citizens. Given that all registries have the same objectives, albeit within their own specific geographic boundaries, and share the same ethical principles, there is, in principle, no reason why such a pan-EU space could not be set up, especially when safeguarded by data sharing agreements. Moreover, the data space would generally only require access to pseudonymized data, consistent with the principles laid out by GDPR to reduce the risks of patient identification. In any case, the fundamental aim of population-based registries is to provide a monitoring and surveillance function for a particular disease; identification or reidentification of patients is not the purpose, and personal identifiers are only used when it is necessary for linking data to obtain the relevant information for selecting cohorts of patients sharing the specific commonalities to which the epidemiological research is addressed. Furthermore, pseudonymized data need never leave the trusted registry space. The processing would be performed entirely within the data space, and only anonymized data would be made available outside the space.

**Figure 3:**
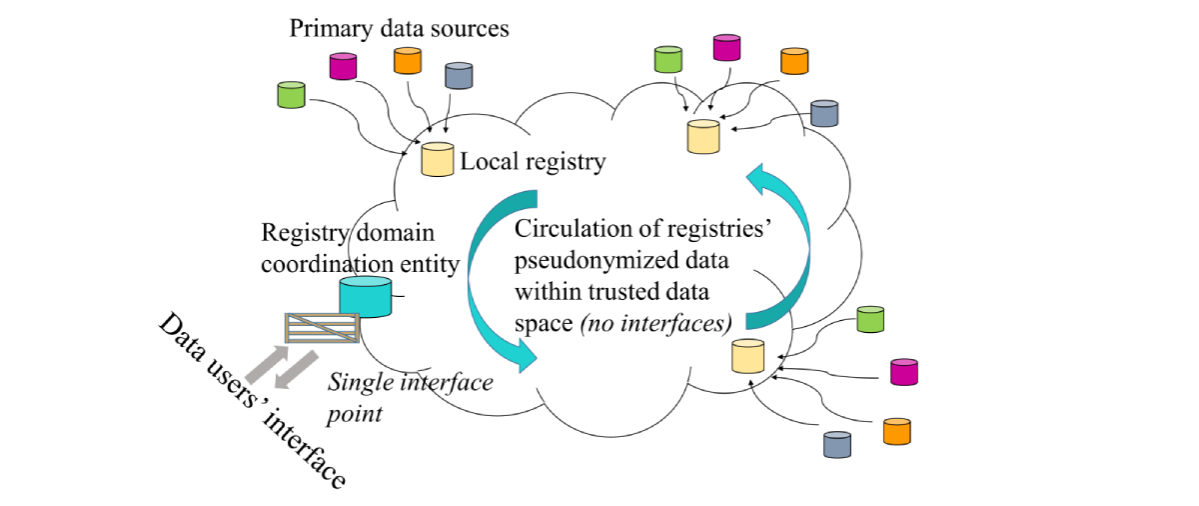
A trusted registry environment or data space for a specific disease domain. The registries within the trusted data space are free to exchange pseudonymized data on the condition that no data leave the space and that the purpose of the exchange is legally and ethically compliant. A single interface point is provided by the coordination entity that works in coordination with all the registries in the data space.

A means for formalizing the network is also needed such that it is recognized as a legal entity. This could be achieved via an administrative and technical entity within the network (and operating in the space, thereby removing a further unnecessary interface and dependence on an external organization) that could manage the budgeting and financial aspects of the network. A registry network with legal status would resolve various difficulties that are becoming increasingly apparent, especially regarding the interface with EHDS, which foresees data nodes as legal entities either at the national level or formally recognized European agencies or bodies (such as the European Centre for Disease Prevention and Control, European Reference Networks, and European Research Infrastructure Consortia). In contrast, registry networks are informal networks of registries that have no legal recognition even though each individual member registry may be a legal entity in its own right. However, with a legal entity status, the administrative entity of the registry network could be formalized as the network’s contact office and serve as the registry domain node in EHDS. Such a contact office may also be able to rationalize the many current access points vis-à-vis the national statistical offices and those offices responsible for providing information to registries. Figure 3 illustrates the benefits of replacing the multiple access points shown in Figure 2 with a single data access point.

## Benefits of the Model

A trusted data space on a disease domain basis, in which registries are able to share pseudonymized data, would transform the current processes of working and considerably facilitate supranational research (such as at the EU level) that can currently only be performed with great difficulty and effort. It would also accelerate the provision of statistical indicators by removing many administrative and bureaucratic hurdles impeding the processes for collating data. An additional potential benefit is that it could provide a safe environment for the data, because the data need never leave the trusted registry space, as is the case in centralized collection points, and thereby motivate the sharing of a rich data set with more variables than are currently included in the minimum data set.

The legal status of the contact office forms a critical element in the network’s business case, as funding for the activities of the network could be provided via administrative and data processing fees to ensure the long-term sustainability of the network. Regarding this aspect, the benefits should be emphasized on the positive feedback mechanism that secondary data use is likely to provide on the quality of data. The more the data are used, the more resources would be available to the network to reinvest in developing more robust processes and tools for verifying the quality of data and extending the set of harmonized variables. Moreover, these resources could in turn be used to support and improve the quality of the primary data feed processes.

A further strength of the model would be to focus the access point to the registry data on a single entity, not only helping to ensure that the data requested from different registries within the same domain are harmonized to the degree necessary for secondary analyses but also allowing a shared view within the community about the ethical aspects and appropriate use of registry data in the incoming data requests. Individual registries within the network would always retain the right to refuse the use of their data in any specific study. Table 1 summarizes the characteristics and advantages of the proposed model over those of the current ENCR model for some of the main data processes.

**Table 1:** Characteristics and advantages of the proposed adapted model compared with the characteristics of the current ENCR^a^ model for some of the main data processes.

Process	ENCR model	Proposed adapted model	Advantages
Data collection	Centralized data collection point	Data remain federated but accessible in data space	All registries have access to other registries’ data Parallelization of centralized tasks, thus avoiding serial data pipeline congestion Improved transparency and verification of data processing Patients’ record-level data remain within the secure data space
Registry interface	Multiple data interfaces between the central data point and registries	No interfaces between registries (data sharing agreements are formalized via initial set of contracts)	Fast availability of data Few administrative processes More rapid derivation of statistical indicators Avoids the need for duplicating data sets and the consequent data integrity issues
Data access	Only the central data point has access to full set of pseudonymized data	All registries can access other registries’ pseudonymized data	More extensive data use, which in turn improves data quality Increased collaborative analyses between registries Deep data pool
Data variables	Data variables are limited to those described in common data set	All data variables are potentially available	High-resolution analyses Facilitation of metadata harmonization (improved visibility of nonstandardized metadata)
Users’ interface	Multiple data interfaces between external data users and registry sources	Single data interface between external data users and registry network	Improved control over data collections and versions, thus avoiding discrepancies in data analyses arising from release of different collections or versions of data
EHDS^b^ interface	No clear interface with EHDS	Interface with EHDS via legal entity representative of the registry network	Well-defined point for data query submissions Fast response times to data queries Improved coordination between registries for handling external data requests

^a^ENCR: European Network of Cancer Registries.

^b^EHDS: European Health Data Space.

There are other benefits that such spaces could bring to the registries’ data feeding processes from the initiating sources, whether from primary sources such as hospitals and clinics or from linkage data (eg, from statistical offices). Registries spend significant amounts of time ensuring consistency and completeness of data [28], both of which are critical processes to remove bias from the data. The time spent in ensuring these particular dimensions of data quality is one of the major contributors to the time delay associated with the provision of validated registry data. Agreeing the common data sets and metadata standards at the European level stand to influence clinical data recording practices across EU to ensure that the common variables are transferred consistently and accurately and to lead to economies of scale by nurturing approaches toward common data quality tools and methodologies. Having 1 point of access to the registry domain would itself facilitate the transfer of death certificate information, which is a piece of critical information required by registries for ensuring data completeness. Therefore, the network could act as a type of data-clearing house, providing a single interface between national statistical offices rather than multiple interfaces that exist currently.

Within the EU at least, there are few reasons to argue against a model that goes in the direction of a trusted data space in which registries are free to exchange pseudonymized data under appropriate and formalized safeguards. It is based on similar arguments as those used to frame the development goals of EHDS but with more justified reason given the very specific aims. The model would provide registries with the autonomy to agree the common data models, metadata, common data sets, and data quality criteria. The latter is essential in any cross-comparison of data and could easily be audited and graded in such a common space, with important ramifications on the trustability of the data in downstream analyses. The model is adaptable to countries at either end of the country income spectrum because it builds on the local registry infrastructure. A study undertaken on the overall costs of CRs for 2010 [29] revealed that costs per inhabitant ranged between €0.03 (US $0.04) and €0.97 (US $1.35), with an average of €0.27 (US $0.37) in comparison with cancer health care of €102 [US $142.8] per EU citizen in 2009 [30]), whereas the costs per registered cancer case ranged between €6 (US $8.4) and €213 (US $298.2), with an average of €50.71 (US $70.9). These figures were comparable with those of registries in the United States. Costs for registries covering large populations were less than those for registries covering small populations, and correspondingly, costs of national registries fared better than regional registries. Increasing expenditure for CRs was associated with the increasing economic wealth of the country, with the greatest proportion of costs being spent on personnel. In view of the health expenditure savings that registry data can bring, the costs are not disproportionate to the total health care costs—at least in the domain of cancer.

## Practical Considerations

A framework allowing such seamless pooling and processing of data, even if only pseudonymized data, would require agreement of national authorities at the EU Member State level despite the provisions laid out in GDPR for processing of sensitive data for reasons relating to the public interest. This would be the first major task to accomplish. However, several aspects will also need to be addressed at a practical level. The first aspect is to identify the means by which a network of legal entities could itself become a legal entity in its own right; having the network agree jointly on terms and implementation would not be sufficient. The solution to this could perhaps be bound together with the legal issues defining a trusted patient registry domain data space. The second aspect relates to the technical aspects of creating a secure EU data space accessible to registries across EU, which may be more difficult to accomplish than a trusted data space housed in and limited to a particular country. The legal and technical aspects may further be compounded by the distinction between EU Member States and European non-EU countries, which are also members of patient registry networks such as ENCR. The third aspect relates to the need for funding, at least initially, before a patient registry domain space could become self-sustaining. A more ambitious endeavor could be to realize a single cross–disease domain patient registry data space, in which resources could be combined to realize a large synergistic technical facility for the development of common processes and tools for data cleaning and validation, without reinventing similar processes and tools on a per patient registry domain basis. There is a risk that a multiplicity of TREs without strong coordination can itself lead to duplicated effort, monopolies on access, and obstructive divergence around data curation [31].

## Conclusions

To overcome the restrictions severely limiting the benefits to public health that could otherwise be achieved by facilitating interregistry integration, a new model is urgently required. The urgency is precipitated by the introduction of GDPR that has changed the context of the former model of collecting national and regional registry data for EU-coordinated actions. A model has been presented that promises to overcome many current hurdles and provide an improved solution to unlock the full potential of integrated registry data by allowing access to the full set of data variables rather than a restricted set. The model builds on the concept of ENCR that has proved to be a successful and viable means for deriving harmonized cancer indicators at the European level but introduces elements critical to a more sustainable and generic framework that could be applied to other NCD domains. The 2 elements that are especially important in this regard are the provision of a trusted data space to facilitate access to pseudonymized data in a secure environment and the creation of a legal entity as an integral part of the registry network serving as the formal single representative of and interface to the network. Regarding the legal entity acting as the interface to the registry network, there is a certain degree of trust required by the registries that can be ensured via appropriate contracts; it would also be critical to ensure stringent technical measures against unauthorized access to the data space and against any possibility of downloading sensitive data. Although both these aspects would require support and formalization at the national level and interregistry contractual agreements regarding the sharing of pseudonymized record-level data, there is, in principle, no reason why this cannot be achieved given the similar aims of local or national registries and explicit agreements at the EU level to tackle the NCD burden.

However, regardless of the specificities of any particular model, the fundamental requirements are to remove the inefficiencies imposed by the various interfaces within the current registry network models and allow a degree of autonomy within a registry domain to provide timely, reliable, and accurate indicators for steering policies to tackle the societal burden of disease and improve the outcomes of patients. In particular, accelerating the availability of data could save considerable costs by feeding back the results of health care measures and programs early in the implementation cycle. The arguments presented are intended to show how a solution for unlocking the resource-rich assets of interconnected patient registries is within reach and how a single interface to these data can most optimally be organized in supranational frameworks such as those existing in Europe. This interface would also provide a controlled means for ensuring access to the harmonized set of European health data in a given disease domain and could serve as an access node within EHDS.

## Ethical Considerations

No ethics approval was applied for since the work described here does not involve research on any of the following: human embryos and fetuses, human cells or tissues, animals, non-EU countries, the environment or health and safety, dual use, or exclusive focus on civil applications. Nor does it contain reference to any personal data and no personal data were collected in the course of the study. Finally, there is no potential for the adverse misuse of the research results here presented. European Commission Horizon 2020 ethical self-assessment [32].

## Abbreviations

CR: cancer registry
EC: European Commission
ECIS: European Cancer Information System
EHDS: European Health Data Space
ENCR: European Network of Cancer Registries
EU: European Union
GDPR: general data protection regulation
NCD: noncommunicable disease
TRE: trusted research environment
WHO: World Health Organization
